# A30 EPITHELIAL FUNCTION OF THE CIRCADIAN CLOCK GENE, BMAL1, IS NECESSARY FOR COLONIC REGENERATION

**DOI:** 10.1093/jcag/gwac036.030

**Published:** 2023-03-07

**Authors:** Z Taleb, M Haireek, K Stokes, H Wang, S Collins, W Khan, P Karpowicz

**Affiliations:** 1 University of Windsor, Windsor; 2 McMaster University; 3 Farncombe Institute, Hamilton, Canada

## Abstract

**Background:**

The circadian clock is a self-sustained molecular oscillator which drives 24-hour physiological rhythms. It consists of the genes *Bmal1* and *Clock* that positively regulate *Cry* and *Per*, their negative regulators, resulting in a 24-hour transcription/translation feedback loop. Shift work, which causes disruptions to 24-hour physiological rhythms, has been shown to lead to an increased incidence of inflammatory bowel disease (IBD). We have previously established that mice lacking the non-redundant circadian regulator, *Bmal1*, exhibit more severe colitis compared to controls.

**Purpose:**

This study aims to investigate the epithelial function of *Bmal1* in colonic regeneration during colitis.

**Method:**

In order to assess the cell-specific role of the clock, we tested the regenerative effects of *Bmal1* in intestinal epithelial tissue using *Vil*^*+/+*^*;Bmal1*^*flox/flox*^ (control) and *Vil*^*Cre/+*^*;Bmal1*^*flox/flox*^ (conditional mutant) mice. Dextran Sulfate Sodium (DSS) was applied to induce acute colitis. Disease progression was evaluated during colitis and during recovery upon removal of DSS treatment. We hypothesized that the absence of a functional circadian clock disrupts effective proliferation and regeneration of intestinal epithelial cells during colitis remission.

**Result(s):**

*Vil* ^*+/+*^ *;Bmal1* ^*flox/flox*^ control and *Vil*^*Cre/+*^*;Bmal1*^*flox/flox*^ conditional mutant mice exhibit no significant differences in disease severity or tissue histopathology during colitis. However, after the removal of DSS, *Vil*^*Cre/+*^*;Bmal1*^*flox/flox*^ conditional mutants show increased total lesions and overall inflammation, decreased crypt density as well as a higher propensity of hyperplastic crypts in the tissue. Regenerative ability of the colon is decreased in conditional mutants: phosphorylated-histone H3 and Ki67 show persistent increases in mitosis and overall proliferation near ulcerated lesions. This suggests that, while controls complete the regenerative response, conditional mutants fail to recover from colitis and show inefficient regeneration when *Bmal1* is disrupted in intestinal epithelial cells.

**Conclusion(s):**

Our results support a critical role for *Bmal1* in intestinal epithelial cells during post-colitis regeneration and recovery.

**Please acknowledge all funding agencies by checking the applicable boxes below:**

CIHR, Other

**Please indicate your source of funding;:**

OGS, Crohn's & Colitis Canada

**Disclosure of Interest:**

None Declared

